# Highly Effective Cow Bone Based Biocomposite for the Sequestration of Organic Pollutant Parameter from Palm Oil Mill Effluent in a Fixed Bed Column Adsorption System

**DOI:** 10.3390/polym14010086

**Published:** 2021-12-27

**Authors:** Adeleke A. Oyekanmi, Mohammed B. Alshammari, Mohamad Nasir Mohamad Ibrahim, Marlia Mohd Hanafiah, Ashraf Y. Elnaggar, Akil Ahmad, Adeleke Teslim Oyediran, Mohd Arif Rosli, Siti Hamidah Mohd Setapar, Nik Norsyahariati Nik Daud, Enas E. Hussein

**Affiliations:** 1 Department of Earth Sciences and Environment, Faculty of Science and Technology, The National University of Malaysia (UKM), Bangi 43600, Selangor, Malaysia; abdulkan2000@yahoo.com; 2 Chemistry Department, College of Sciences and Humanities, Prince Sattam Bin Abdulaziz University, Al-Kharj 11942, Saudi Arabia; m.alshammari@psau.edu.sa; 3 School of Chemical Sciences, Universiti Sains Malaysia, Penang 11800, Malaysia; mnm@usm.my; 4 Centre for Tropical Climate Change, Institute of Climate Change, Universiti Kebangsaan Malaysia, Bangi 43600, Malaysia; 5 Department of Food Nutrition Science, College of Science, Taif University, P.O. Box 11099, Taif 219944, Saudi Arabia; aynaggar@tu.edu.sa; 6 Centre of Lipids Engineering and Applied Research, Universiti Teknologi Malaysia, Bahru 81310, Malaysia; siti-h@utm.my; 7 Department of Geoscience, Institute of Hydrocarbon Recovery, Univerisiti Teknologi Petronas (UTP), Perak 32610, Malaysia; tessylink@yahoo.com; 8 Faculty of Engineering Technology, Universiti Tun Hussein Onn Malaysia, Parit Raja 86400, Malaysia; mohdarif@uthm.edu.my; 9 Department of Civil Engineering, Faculty of Engineering, Universiti Putra Malaysia, Kuala Lumpur 43400, Malaysia; niknor@upm.edu.my; 10 National Water Research Centre, P.O. Box 74, Shubra, El-Kheima 13411, Egypt; enas_el-sayed@nwrc.gov.eg

**Keywords:** biosorbent, biocomposites, organic pollutants, palm oil mill effluent, fixed bed column

## Abstract

The reduction of chemical oxygen demand (COD) from palm oil mill effluent (POME) is very significant to ensure aquatic protection and the environment. Continuous adsorption of COD in a fixed bed column can be an effective treatment process for its reduction prior to discharge. Adsorption capacity of bone derived biocomposite synthesized from fresh cow bones, zeolite, and coconut shells for the reduction in the organic pollutant parameter was investigated in this study in a fixed bed column. The effect of influent flow rate (1.4, 2.0, and 2.6 mL/min) was determined at an influent pH 7. The optimum bed capacity on the fabricated composite of surface area of 251.9669 m^2^/g was obtained at 1.4 mL/min at breakthrough time of 5.15 h influent POME concentration. The experimental data were fitted to Thomas, Adams–Bohart, and Yoon–Nelson models fixed bed adsorption models. It was revealed that the results fitted well to the Adams Bohart model with a correlation coefficient of R^2^ > 0.96 at different influent concentration. Adsorption rate constant was observed to increase at lower flow rate influent concentration, resulting in longer empty bed contact time (EBCT) for the mass transfer zone of the column to reach the outlet of the effluent concentration. In general, the overall kinetics of adsorption indicated that the reduction in COD from POME using a bone-biocomposite was effective at the initial stage of adsorption. The pore diffusion model better described the breakthrough characteristics for COD reduction with high correlation coefficient. Shorter breakthrough time compared to EBCT before regeneration indicated that the bone composite was suitable and effective for the reduction in COD from POME using fixed bed column adsorption.

## 1. Introduction

The palm oil mill industry is the major agro-industrial sector in Malaysia. During the processing of palm oil, the production of the primary products generates different types of waste such as empty fruit bunch, (EFB), oil palm shells (OPS), and palm oil mill effluent (POME) [1]. According to the statistics, the palm oil industry generates about 48–72 million tonnes of POME per year in countries such as Malaysia and Indonesia [2]. Chin et al. [3] stated that the palm oil mill industry is the major contributor of industrial pollution in Malaysia. The POME produced increases sludge generation and moisture content enriched with organic matter. The organic pollutants of palm oil based waste can promote microbial growth, affecting the flora and fauna of the water ways [4,5]. Furthermore, Tan et al. [6] reported that the acidic, vicious, and obnoxious odour of POME increased the potential of environmental pollution. POME contains between 95–975 moisture, 4–5% total solid, 2–4% suspended solids including an average of 19,610 mg/L of total suspended solids (TSS), 25,000 mg/L of biochemical oxygen demand (BOD) and 55,250 mg/L of chemical oxygen demand (COD) [7]. According to the stringent discharge limit set by the Malaysian Department of Environment (DOE) to ensure environmental safety and sustainability through POME treatment before discharge, the average COD in POME must not exceed 1000 mg/L [8]. The high concentration of COD in POME is attributed to the high contents of organic matter and carotene pigment as well as other compounds such as polyphenol compounds, polyalcohol, tannin, and melanoid, which are generated from the fresh fruit bunches during the sterilization process [9,10,11]. Consequently, COD is the major pollutant parameter in POME that affects aquatic respiration and the environment if not properly treated before discharge [5,12], This physicochemical parameter must be reduced to appreciable low concentration due to the hazardous effect to human, animals and the ecosystem. The deposition of POME must comply with the prevailing effluent discharge standard to ensure environmental sustainability.

However, the high amount of effluent generated in the palm oil mill industry has increased research methods to minimize the effect of water pollution. Some physico-chemical treatment process has been widely reported for the treatment of POME, some of which include reverse osmosis [13], coagulation-flocculation process [14], advanced oxidation [15], photocatalysis [16], ultrafiltration membranes [17], and biological processes treatment.

The treatment efficiency of each method varies depending on the technology employed. The effectiveness of the treatment system is the ability of the treated effluent to ensure safe discharge for the protection of the aquatic population and the environment [18,19]. POME is a high strength wastewater, the concentration of COD could be as high as 100,000 mg/L, which is extremely hazardous to the environment and the receiving water [20]. The reduction in COD and NH_3_–N in a batch adsorption mode has been reported in our previous studies as a pioneer research [21]. However, the reduction in the COD pollutant parameter in POME in a fixed bed adsorption column study has not been reported in the literature. The previous studies from our search investigation studied the fixed bed adsorption of POME for the removal of colour and COD using an anion based resin [22,23]. The application of a composite adsorbent nowadays is receiving attention due to its potential for a higher adsorption capacity of the pollutant parameters. The removal of organic pollutants from dyes has been reported using a chitosan supported graphene oxide-hydroxyapatite composite [24], and a silica–sand starch composite was synthesized and applied for the removal of heavy metals and dyes from aqueous solution [25]. Magnetic carbon biomass from seeds of *Moringa oleifera* MnFe_2_O_4_ has been applied for dye removal from aqueous solution [26]. Similarly, an alginate activated carbon composite has been reported for the removal of methylene blue from an aqueous solution in a fixed bed adsorption study [27]. Additionally, the adsorption of total organic acid onto an alginate/clay hybrid composite in a fixed bed continuous column has been reported by Edathil et al. [28]. In addition, the functional properties of adsorbents fabricated as composites can improve adsorption capacities significantly due to enhanced surface area, chemical properties, and mechanical stability [29]. The removal of organic solutes from adsorbate largely depends on the hydrophobicity of the material [30]. The surface of carbon is hydrophobic with pore sizes that make it more suitable for the adsorption of organic substances [31,32]. The dispersed trace organic micropollutant molecules are strung together by the influence of H-bonding to form hydrophobic clusters that are attached on the hydrophobic membrane surface and are known to improve the adsorption affinity [33]. The efficiency of organic pollutants from POME using a novel bone-composite in a batch adsorption has been reported in our previous study [21]. However, the adsorption of COD from POME using bone derived composite has not been reported in the literature.

This work was conducted to investigate the fixed bed adsorption capacity of a bone-composite for COD reduction from POME via a column lab scale study. The effects of varying flow rates on the breakthrough characteristics of the adsorption system were examined. The breakthrough characteristics of the fixed-bed adsorption system for the reduction in COD concentration from POME was investigated using the Adams–Bohart, Thomas, and Yoon–Nelson models.

## 2. Materials and Methods

### 2.1. Collection of POME

An approximately 20 L sample of POME was obtained from the processing mill plant of the palm oil production unit located at Kilang Sawit PPNJ (2°10 I 23.6 I N, 103°, 28 I 49.3 I E) Malaysia. The effluents were obtained at an average temperature of 95 °C. The method of storage and preservation was accordance with the international standard procedure [34]. Prior to investigation of the column adsorption study, the initial concentration of the physico-chemical parameters was obtained according to standard methods.

### 2.2. Preparation of Bone-Biocomposite Adsorbent

The preparation and characterization including the morphological and chemical composition of the composite adsorbent derived from the activated cow bone powder, activated coconut shells, and zeolite were undertaken according to our previous study with slight modifications [35]. Fresh cow bones was obtained at the Suan Hin Mini local stall market located in Penang, Malaysia. The bones were thoroughly washed with deionized water and oven-dried for 48 h to reduce internal moisture content. The precursor was heated in the furnace under a controlled temperature of 700 °C for 5 h. Coconut shells was obtained from Nibong Tebal Penang and used to prepare the activated carbon. The precursor was washed several times and dried for 24 h at 105 °C. About 10 g each of dried shells were immersed in 85 wt.% of a solution of phosphoric acid (0.1–1.5 g/g acid:precursor ratio) was supplied by Rada Synergy Sdn Bhd, Puchong, Malaysia. 

The mixture was heated in a (30 φ × 360) tubular glass quartz at 150 °C for 2 h at a heating rate of 10 °C/min, after which the mixture was heated at 400–800 °C at a heating rate of 10 °C/min within 1–4 h at a flow rate of 250 mL/min [36]. The samples were cooled to room temperature and washed with hot deionized water until neutral pH was obtained. Next, the sample was dried at 120 °C for 3 h. The activated carbon obtained was modified with nitric acid solution and stirred between 3 and 5 h at 90 °C. The activated carbon was later dried at 120 °C for 3 h in a 100 mL of 1–10 mol/L nitric acid aqueous solution (Rada Synergy Sdn Bhd, Puchong, Malaysia).

The modified sample was washed with hot distilled water until the pH value of the filtering solution became neutral. Natural zeolite in the range of 3 to 6 mm was supplied by YM Multi Trading Company, Selangor, Malaysia. Prior to the investigation, the zeolites were washed thoroughly with distilled water to remove surface impurities and then dried for 24 h at 105 °C in a furnace (Memmert, Schwabach, Germany). Afterward, the dried granular zeolites were crushed using crusher machines (Retsch, Haan, Germany) and sieved to obtain an average sample size of 150 µm. The mixture composition of the adsorbents for the synthesis of the biocomposite was adopted from a previous study [21].

The surface area of the composite was investigated in the present study for the design of the fixed bed apparatus for the fixed bed column (Table 1). The Brunauer–Emmett–Teller (BET) and t-plot methods were conducted for the determination of the surface area of the composite while the Barrett–Joynes–Halenda (BJH) was used for the estimation of the distribution of the pore size. The surface area properties of the composite for the continuous fixed bed adsorption study is presented in Table 2. Finally, the surface charge of the prepared composite was achieved at different pH values by conducting point of zero charge analysis. The pH of the sample was adjusted using hydrochloric acid (2.5 M) supplied by Brenntag Sdn. Bhd. Selangor Malaysia and sodium hydroxide (2.5 M) solutions (Brenntag Sdn. Bhd. Selangor Malaysia). This analysis provided a clear description of the surface chemistry of the composite as well as an understanding of the adsorption mechanism.

### 2.3. Fixed Bed Adsorption Parameters

**Figure 1:**
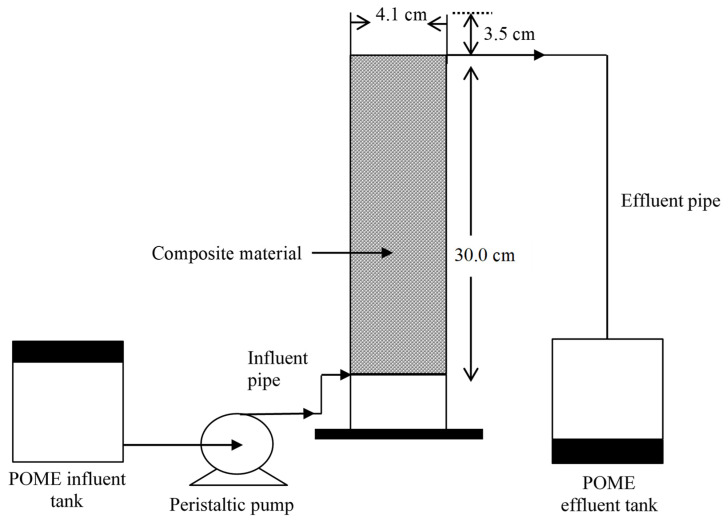
Schematic of the fixed bed column study.

A schematic of the fixed bed adsorption system used for the adsorption of the granular composite is illustrated in Figure 1. A Pyrex glass cylinder with a 4.1 cm inner diameter and 30 cm bed depth was used to conduct a continuous flow adsorption study. The other evaluated parameters for the design of the breakthrough curve was used for the design of EBCT on the basis of the nature of the composite adsorbent. Other design considerations that were applied for the design of the fixed bed column and continuous bed adsorption models are presented in Table 1. The surface area of the column was evaluated from the size of the inner diameter. The weight of the adsorbent was measured and was filled in the column to the mark of the free board. The BET surface area and pore size of the composite in the packed column is shown in Table 2. Samples were collected at intervals and analysed using the closed reflux method for the effluent concentration of COD. The column study was conducted in triplicate to obtain a repeatable result.

### 2.4. Method of Adsorption Study

Continuous flow adsorption studies were conducted in a glass column with a 0.041 m internal diameter and a 0.30 m height of the column excluding the freeboard. A series of experiments were conducted with an influent concentration of POME in the packed composite column. In the column setup, a stainless steel was inserted to the bottom of the column and above the sieve was a layer of glass wool to prevent the loss of the adsorbent. A master flex peristaltic pump (Model Master flex Cole-Parmer Instrument Co.,Vernon Hills, IL, USA) was used for the upward movement of the effluent through the column at the designed flow rate and empty bed contact time (EBCT). The effluent was placed in a tank and pumped in the upward direction into the column. The solution was mixed with the composite until it attained the saturation point. Effects of process parameters such as flow rates (1.4, 2.0, and 2.6 mL/min) and other parameters were kept constant according to Table 1. Samples were then collected at various time intervals from the upper of the column and were investigated to determine the COD in the effluent concentration of POME.

### 2.5. Breakthrough Characteristics

The time taken to reach the breakthrough at different influent concentration of flow rates was analysed. The results of the investigation were plotted to achieve the breakthrough curves to understand the behaviour of the composite adsorbent at different time intervals under different conditions of continuous flow adsorption. The investigation was expressed in terms of the effluent concentration of adsorbate with time ($C_{t}$/$C_{O}$ against t). The equilibrium adsorbate in the column ($q_{eq}$) was obtained at 50% breakthrough concentration. The experiments were conducted at the optimum pH obtained from the batch study and at room temperature (30 ± 1) °C.

The breakthrough time (t), is refereed as the time at which the effluent COD concentration (*C_t_/C*_0_) exceeds 0.05, which is determined from the breakthrough curves. The breakthrough characteristics of the bone-composite under various adsorption conditions were fitted to the Thomas, Adams–Bohart, and Yoon–Nelson models, which were evaluated from the slopes and intercepts of the linearized equations.

#### 2.5.1. Thomas Model

The Thomas model is widely applied for the determination of the rate constant for the maximum adsorption capacity of an adsorbate in a column study. The derivative of the model is based on the assumption of Langmuir kinetics of adsorption-desorption [37]. The Thomas equation obeys the second order reversible reaction kinetics.

The equation is expressed as:

(1) $$\frac{c}{c_{o}}=\frac{1}{1+\exp\;K_{\frac{TH}{Q}\;\left(q_{o\;}x-C_{o\;Veff}\right)}}$$

where

$K_{TH}\;$= Thomas rate constant;
*Q* = flow rate;
$q_{o}$ = maximum solid-phase concentration of the solute;
x = amount of adsorbent in the column; and
$V_{eff}$ = volume of effluent.

In linearized form, the equation can be represented as:

(2) $$\ln\left(\frac{C_{O}}{C_{e}}-1\right)=\frac{K_{TH}\;q_{o}x}{Q}-\frac{K_{TH}\;C_{O}V_{eff}}{Q}$$

A graph of $\ln\left(\frac{C_{O}}{C_{e}}-1\right)$ is plotted against time, t.

The expression of $\frac{K_{TH}\;C_{O}V_{eff}}{Q}$ is equivalent to the slope of the linear regression while $\frac{K_{TH}\;q_{o}x}{Q}\;$ is equivalent to the intercept.

#### 2.5.2. Adams–Bohart Model

The Adams–Bohart model was used to describe the relationship between $\frac{C}{Co}$ and time for the adsorption of in a fixed bed activated carbon [38]. The model was initially proposed for gases and later to liquids by the replacement of the terms in pressure by concentration. The model is developed based on the surface reaction and the prediction that equilibrium is not instantaneous. Hence, it was postulated that the rate of adsorption is proportional to the remaining capacity of the adsorbent and the adsorbate concentration, the description of adsorption using one adsorbate is better described using Adam- Bohart model [39]. The equation is given by:

(3) $$\ln\left(\frac{C_{e}}{C_{O}}\right)=KC_{O}t-\;KN_{o}\frac{Z}{U}$$

where,

$C_{O}$ = the initial concentration;
$C_{e}$ = instantaneous concentration of the solute (g/L);
K = kinetic constant (L/g/min);
$N_{o}\;$= adsorption capacity;
Z = column bed depth; and A0
U = speed of gas out (m/s).

A linear plot of $\ln\left(\frac{C_{t}}{C_{O}}\right)$ against time gives a slope of *No* and intercept of K.

#### 2.5.3. Yoon–Nelson Model

The Yoon–Nelson model is an extrapolation model that utilizes the experimental data to evaluate parameters that are computed into the model. The model assumes that the rate of decrease in the probability of adsorbate adsorption for the adsorbate molecule is proportional to the probability of the adsorbate and the breakthrough curve of the adsorbent [40].

The model in a linear form is expressed as:

(4) $$\ln\left(\frac{C_{t}}{C_{O}-C_{t}}\right)=\mathrm{Kynt}-\lambda \mathrm{Kyn}$$

where

Kyn (Min-1) = rate constant; and
λ= time required for breakthrough of 50% adsorbate.

The values of Kyn and λ is equal to the intercept and slope, respectively.

### 2.6. Desorption Study

The application of regeneration study was conducted to investigate the ability of the spent adsorbent in the column to be reused for further adsorption study. However, to achieve this purpose, a solution of sodium hydroxide was used as the regenerating solution. Before the regeneration process, the column was drained of the POME in the column, after which the spent adsorbent was regenerated using a 0.5 M NaOH solution supplied by Brenntag Sdn. Bhd. Selangor Malaysia at the optimum flow rate influent concentration, which was obtained from the column study. The adsorption bed was considered exhausted when the concentration of effluent of the column outlet is almost equal to the concentration at the influent [41]. The concentration of COD removal from the optimum flow rate influent concentration was used to determine the regeneration study of the spent adsorbent. The adsorbent was considered regenerated if the concentration of COD was close or equal to zero. After the regeneration of the adsorbent was achieved, the column was washed with deionized water until pH 7 was achieved. After the equilibrium condition was attained, the adsorption study was conducted on the regenerated adsorbent. All experiments were conducted in duplicate and the average percentage removal was achieved.

The point of zero charge (pHpzc) determines the point at which the interfacial region of a material is electrically neutral. The experiment to determine the pHpzc of the composite was carried out using the 11 point pH measurement as adopted by Chechinel et al. [42]. A sample of 0.025 g of adsorbent weight of the composite was placed in a 100 mL conical flask, and 25 mL of deionized water was added to each of the flasks at different initial pH conditions. The pH was adjusted using 0.1 M NaOH and HNO_3_. The samples were agitated at 120 rpm for 24 h at 25 °C. At the end of the contact time, the final pH of each of the samples was measured. A plot of the final pH against the initial pH was produced from the obtained values. The point at which the plot of the final pH intersects with the plot of the initial pH value was recorded as the pHpzc.

## 3. Results and Discussion

### 3.1. Influence of Solution pH and Point Zero Charge (pHpzc)

**Figure 2:**
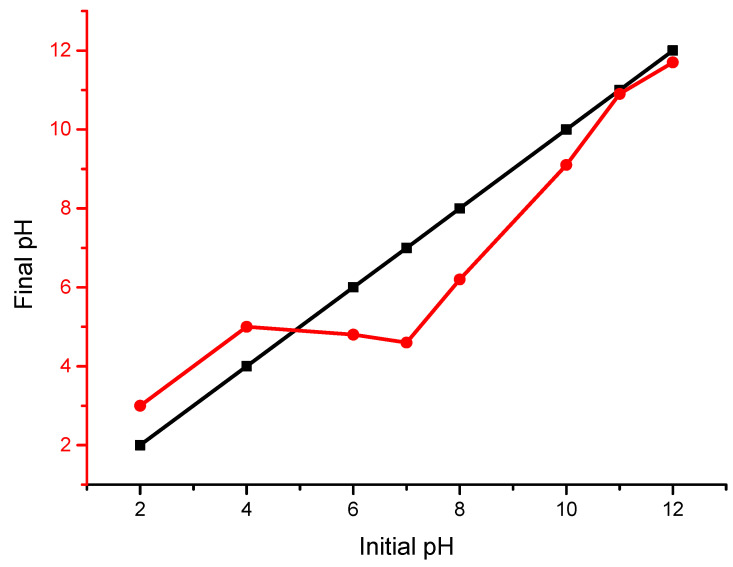
Initial vs. final pH plot for the determination of the pHpzc of the composite in the packed column.

The pH of the solution plays an important role in thee adsorption study and was used to determine the surface charge on the active site of the composite, which influenced the adsorption of the analytes. The point of zero charge is the pH at which the surface of the adsorbent has no electric charge. It was observed that the pHpzc was 5.28 in Figure 2, which signified the presence of acidic sites with a negatively charged surface. After this condition, the pH of the composite was below the pHpzc as the pH increased, which indicated a positively charged surface. At the pHpzc, the surface of the composite was acidic, which indicated that the surface charge density decreased with an increase in the pH of the supernatant of the POME (pH > pHpzc), indicating that the acid surface was attributed to the presence of the carboxylic group on the surface [43]. Hence, the electrostatic repulsion between the positively charged ions of POME and the composite was lower, resulting in the increase in the adsorption of cations of POME. Since the adsorption of solutes of POME is more favourable on the acidic surface, it therefore implies that the pollutants of POME can be effectively adsorbed onto the acidic sites of the composite since the acidic sites were negatively charged.

The acidic sites of the composite formed due to the reaction between the hydroxyl groups on the composite surface and the H^+^ ion in the POME [44]. Under the acidic condition, the interaction between the hydroxyl groups of the composite and the anionic sites of POME enhanced the chemisorption of the organic pollutants of POME onto the composite surface. It was reported in the study by Chechinel et al. [42] that cow bone adsorbents had remarkable potential for the removal of pollutants from wastewater. It was revealed that activated cow bones under an acidic condition are composed of a negatively charged surface. The effectiveness of the adsorption process was attributed to the acidic condition of the analyte. It was indicated that pHpzc was obtained at 4. In the study of the adsorption of fluoride from an aqueous solution using an aluminium based impregnated carbon, Ramos et al. [45] observed that aluminium precipitates as Al(OH)_3_ in the aqueous solution and influenced the adsorption of fluoride from the solution. This was achieved at an optimum pH of 3.5. Furthermore, it was revealed that calcium ion precipitates as Ca(OH)_2_ in the supernatant [46], suggesting that the reduction in COD from POME was influenced by the precipitation of Ca(OH)_2_ as a component mixture in the composite. Therefore, the effect of the acidic condition of the composite and the abundance of Ca(OH)_2_ from the cow bone precursor in the composite had a profound influence on the adsorption of COD from POME in the fixed bed continuous column adsorption.

### 3.2. Effect of the Influent Flow Rate Concentration

**Figure 3:**
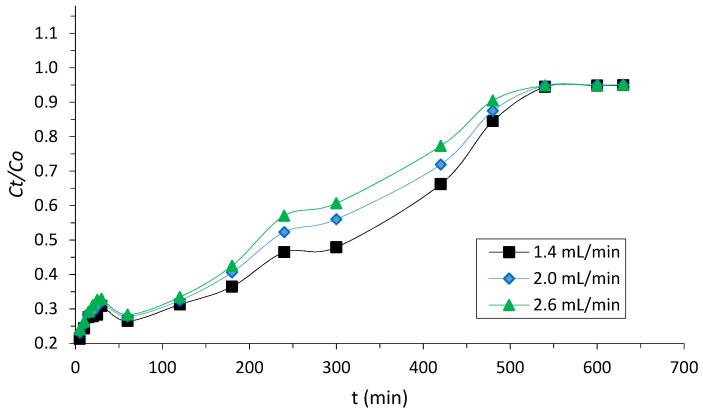
Breakthrough curve at different flow rate influent concentrations.

The effect of the influent flow rate on the reduction of COD from the adsorbate onto the composite adsorbent was investigated at different flow rates (1.4, 2, and 2.6 mL/min). The experiments were conducted at a fixed condition of bed height (30 cm) and pH (10). The pH of the adsorbate was adjusted using diluted H_2_SO_4_ and NaOH. The breakthrough curves is illustrated in Figure 3.

In view of the results obtained, at a lower flow rate influent concentration, there was a longer EBCT for the mass transfer zone of the column to reach the outlet of the effluent concentration. This trend indicates that the saturation time increased in the packed column, resulting in the increase in the mass transfer zone between the composite adsorbent and the POME [47]. At higher flow rates, there was evidence of early breakthrough with steeper curves, resulting in less reduction of the COD concentration in the POME. As the flow rate decreased, the time to reach breakthrough increased. As the residence time increased, diffusion into the pores of the composite was achieved, resulting in higher adsorption capacity of the composite for the removal of COD from POME. It was revealed that the optimum reduction in the COD concentration occurred at the influent flow rate concentration of 1.4 mL/min (Figure 3). However, evidence of high adsorption capacity at the optimum flow rate has been reported in the literature where at lower flow rates, there is the tendency of the pollutant parameter to be adsorbed on the active sites of the composite in the mass transfer zone [48]. In this study, the EBCT was evaluated from the rate of bed volume with flow rate (5).

(5) $$\;\;\;\mathrm{EBCT}=\frac{\mathrm{Bed}\;\mathrm{volume}}{\mathrm{Flow}\;\mathrm{rate}}$$

It can be seen that at a higher flow rate, a lower EBCT was achieved. It was shown that at a lower EBCT, there was a lower diffusion process of the solute–adsorbent interaction, and this trend resulted in a lower adsorption capacity [31,49]. From the results of the investigation, it was revealed that the composite packed column enhanced the reduction in COD more at a lower flow rate influent concentration. The longer breakthrough time of 5.15 h was obtained at the optimum flow rate. The retention time became shorter with an increase in the velocity of the influent concentration. It is apparent that at the influent flow rate of 2 mL/min, the breakthrough time was achieved at 4.45 h. It was observed that the breakthrough time was 4.26 h at 2.6 mL/min. Therefore, it revealed that the breakthrough time was the lowest at the highest flow rate influent concentration. This phenomenon was due to a shorter contact time between the interaction of the solute and the composite adsorbent in the packed column. This resulted in less diffusion of the solutes into the pores of the composite. This trend was observed in the previous studies [50,51].

### 3.3. Packed Column Adsorption Models

The adsorption capacity of the bone-composite was investigated in a fixed bed adsorption column. The solute in the adsorbate solution moved through the composite in the column. This resulted in the increase in the adsorption zone until it reached the effluent concentration. The time taken for the influent concentration to reach breakthrough is referred to as the breakthrough time. The point at which the outlet concentration reached the inlet concentration is referred to as the exhaustion time. The adsorption mechanism of the COD reduction on the packed composite was analysed using the Thomas, Adam–Bohart, and Yoon–Nelson models. The optimum flow rate obtained from the breakthrough curve at the concentration ratio *C_t_*/*C_o_* > 0.1 until *C_t_*/*C_o_* > 0.99. The capacity of the column for the reduction in COD was evaluated by the models.

#### 3.3.1. Thomas Model

**Figure 4:**
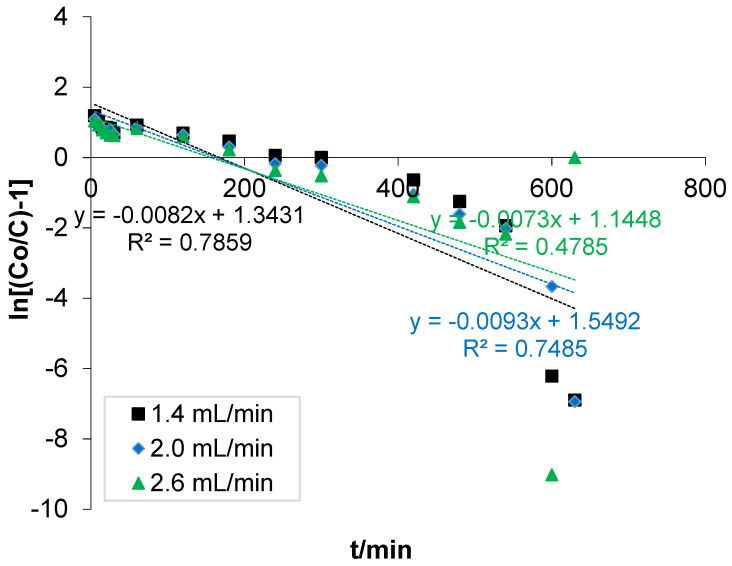
Linear plot of fitted data to the Thomas model.

The data obtained from the fixed bed adsorption for the optimum flow rate influent concentration were fitted to the Thomas model (Figure 4). The model is one of the most widely used to describe the adsorption process behaviour in fixed-bed columns. The model, which is derived from the second–order kinetics, assumes that adsorption is dominated by mass transfer at the interface and the chemical reaction [52]. It is generally known that the slight reduction in correlation coefficient values (R^2^) indicated that the main limitation is that its derivation is based on a second-order kinetics and considers that adsorption is not limited by the chemical reaction, but is controlled by the mass transfer at the interface [53].

It was revealed from the fitted data that the model had the best R^2^ at the least flow rate. It was indicated that as the flow rate increased, the value of R^2^ decreased, suggesting that bone-composite effectively reduced COD in the POME at a low flow rate as a result of the interaction and diffusion of solutes in the pores of the composite.

#### 3.3.2. Adam–Bohart Model

**Figure 5:**
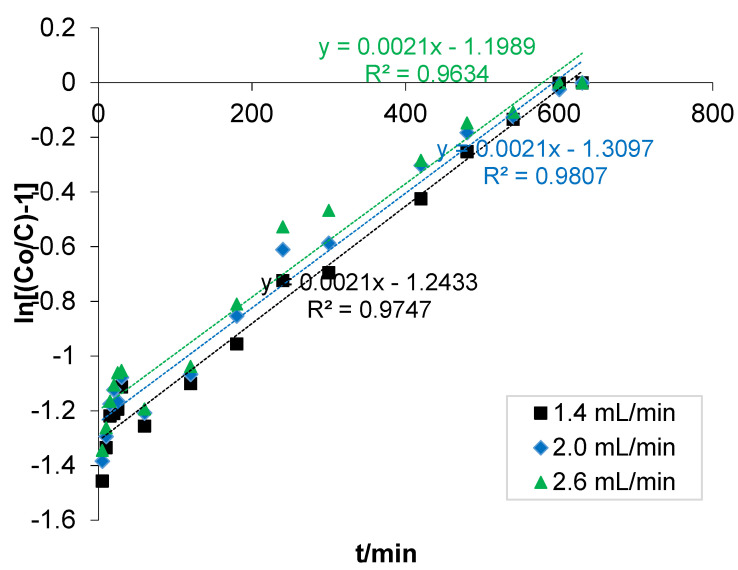
Linear plot of the Adam–Bohart model.

In order to assess the breakthrough characteristics and the adsorption capacity of the composite, further reduction in COD in the packed column was analysed. Generally, the Adams–Bohart adsorption model was applied to the experimental data for the description of the initial part of the breakthrough curve. The values of ln (*C_t_*/*C_o_*) were plotted against time (t). The result was used to evaluate the maximum adsorption capacity (NO) and the mass transfer coefficient (K_ab_). The linear plot of the fitted data to the model is presented in Figure 5.

The N_o_ and K_ab_ were calculated from the intercept and slope of the fitted values. The adsorption capacity (No) was 96,420 mg/L and the kinetic constant (K_ab_) was 4.7 × 10^−8^ L/mg. However, from the results of the investigation, a higher R2 (0.95007) was achieved. Although the correlation coefficient for the two models had close fitted values, it was observed that the N_o_ was significantly higher with the prediction of the model. The adsorption capacity increased as the influent concentration increased, indicating that the overall system of adsorption is dominated by mass transfer and diffusion of the solute into the pores of the packed composite [54]. A similar observation was reported in the previous studies [55,56].

#### 3.3.3. Yoon–Nelson Model

**Figure 6:**
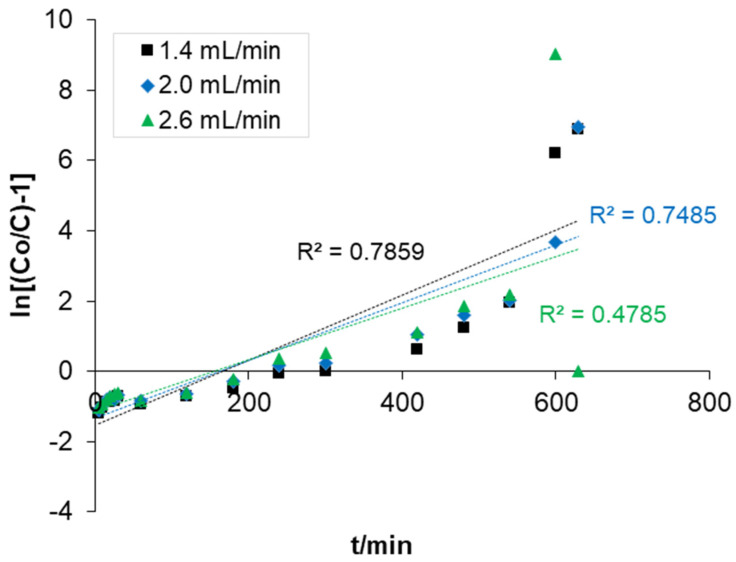
Linear plot of the Yoon–Nelson model.

The Yoon–Nelson model is a fixed bed model used to predict the exhaustion time and the behaviour of the adsorption process for a given adsorbate concentration [57]. The model assumes that the rate of probability decline of adsorption for each adsorbate molecule is proportional to the adsorbate adsorption and breakthrough [58]. A linear plot of ln[(*C_o_*/*C*) − 1] against sampling time (t) and the values of ι and kYN were determined from the intercept and slope of the plot, respectively. The variation in the linear graph for adsorption of COD ions onto the bone-composite for different flow rates is shown in Figure 6. As can be seen, the flow rate increased from 1.4 to 2.6 mL/min at a constant bed depth of 30.0 cm, and the rate constant (KYN) increased with little significance while the time required for 50% breakthrough decreased. The value of the R^2^ obtained from the fitted experimental data to the model at different flow rates indicated that earlier breakthrough was reached at a higher flow rate and at less residence time of the solute to diffuse through the length of the sorption column [22]. The results obtained agreed with previous work carried out by [23].

Generally, irrespective of the influent flow rate determined by the operational condition, it was revealed that lower correlation coefficients were obtained from the data fitted to the Thomas and Yoon–Nelson models. The value of the correlation coefficient values for the Thomas and Yoon–Nelson models was the lowest for a given experimental condition, while the highest R^2^ was obtained for the data fitted to the Adams–Bohart model. Thus, it could be concluded that the Adam–Bohart model better describes the process of COD reduction in the fixed-bed column.

### 3.4. Desorption of Spent Composite

**Figure 7:**
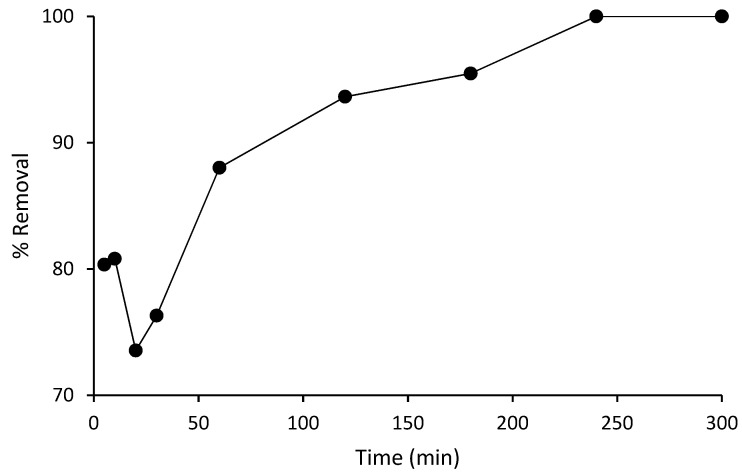
Breakthrough curve after desorption.

To ensure that the prepared composite adsorbent is viable for adsorption purposes for the purpose of reuse, the ability to regenerate the spent adsorbent is essential to enhance the adsorption technology. The adsorbent can be reused after complete desorption of the solutes from the composite by desorption analysis using a regenerating solution for desorption of the solutes from the composite. In this work, the regenerating study was carried using 0.5 M NaOH solution as the eluting agent. The solution was passed into the column using the optimum flow rate influent concentration obtained from the breakthrough curve in the fixed bed adsorption. Desorption of the solute was achieved after the COD concentration was completely desorbed from the spent composite. The experimental data obtained from the regeneration study was used to plot the graph of percent (%) removal against time (t). It was revealed that significant COD removal was achieved after the regeneration of the spent composite until 6 h breakthrough contact time. The result is presented in Figure 7.

**Figure 8:**
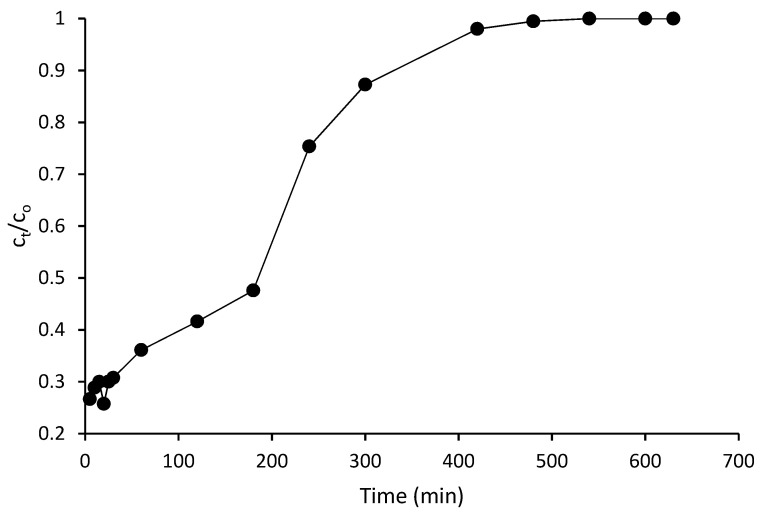
Breakthrough curve after regeneration of the composite.

It was observed that the time for the 50% breakthrough was achieved at 3.17 h, as shown in Figure 8. The value of the breakthrough time was shorter than the breakthrough contact time achieved at the optimum influent concentration before regeneration. In summary, the prepared composite adsorbent developed from the activated coconut shell carbon, activated cow bone powder, and zeolite was found to be very effective for the reduction in COD in the POME. The effect of the influent flow rate on the breakthrough curve characteristics of COD was studied. The 0.5 M NaOH solution was chosen for desorption purposes. It was observed that the spent composite adsorbent was regenerated at a lower contact time than the exhaustion time. Therefore, the investigation revealed the suitability of the composite adsorbent for the adsorption of the organic pollutant parameter and the ability of the composite to be reused, indicating its economic benefit for the treatment of high strength POME.

## 4. Fixed Bed Adsorption Mechanism

The mechanism of reduction in COD through the bone-biocomposite was influenced by various factors such as the pH of the solution, surface charge of the composites, pore diffusion, and adsorption kinetics. The pH of the solution is an important parameter and plays a key role in the adsorption process and significantly determines the surface charge of the composite materials that influence the adsorption of organic pollutants of COD from the POME solution. Maximum reduction in COD was observed in the acidic medium. This might be due to the acidic environment of working pH where the interaction between the hydroxyl groups of the bone-biocomposite and the anionic sites of POME can enhance the chemisorption of the organic pollutants (COD) of POME onto the composite surface. From the results, it was observed that electrostatic repulsion between the positively charged ions of POME and the composite was lower, resulting in the increase in the adsorption of cations of POME. Hence, a reduction in the organic analytes of POME was more favourable on the acidic surface of the composites. Therefore, it implies that the pollutants of POME can be effectively reduced onto the acidic sites of the composite since the acidic sites were negatively charged. From the pore diffusion model, the study well described the breakthrough characteristics for COD reduction with a high correlation coefficient. However, a shorter breakthrough time compared to EBCT before regeneration indicated that the bone composite was suitable and effective for the reduction in COD from POME using fixed bed column adsorption. From the study, the overall adsorption process indicated that the reduction in COD from POME using the bone-biocomposite was effective at the initial stage of adsorption.

## 5. Conclusions

This work was devoted to assessing the capability of fixed-bed column adsorption to predict the breakthrough characteristics by using three models, namely, Thomas, Adam–Bohart, and Yoon–Nelson. The suggested optimum bed capacity on the fabricated composite of the surface area of 251.9669 m^2^/g was obtained at 1.4 mL/min at a breakthrough time of 5.15 h. Results indicated that the breakthrough time, exhaustion time, and adsorption capacity at breakthrough increased as the flow rate decreased. Experimental breakthrough curves were observed to be in good agreement with the theoretical values by the Adam–Bohart and Yoon–Nelson models, which were validated by the correlation coefficient values (R^2^). The results revealed that the bone-composite was an effective adsorbent and can be applied in a large scale fixed bed column for the removal of organic pollutant parameters from palm oil mill effluent. Furthermore, the bone-composite exhibited high reusability potential. Adsorption capacity of the composite adsorbent achieved 99.47% removal of COD from POME in the continuous flow packed column after regeneration at 4.1 h until 5 h, revealing rapid removal efficiency of the composite for organic pollutant remediation. It was indicated that the spent composite could be regenerated using 0.5 M NaOH solution with a slightly lower adsorption capacity compared to the fresh composite. In general, the bone-composite can be utilized to model the industrial behaviour of porous adsorbents when the resistances to mass transport by the diffusion of the adsorbate are significant. Nevertheless, future studies are required in experimental and modelling at varying bed depths, accounting explicitly for the diffusion phenomena of the bone-composite.

## Figures and Tables

**Table 1:** Design of fixed bed adsorption parameters.

Parameter	Unit	Value
Diameter, D	m	0.041
Surface Area of Column, A	m^2^	0.0013
Height of Media, H	m	0.30
Volume of Column, V	m^3^	0.00040
Density of Column, ρ	kg/m^3^	405.00
Porosity, _ε_	%	41.90
V porosity, V_ε_	m^3^	0.000167
Mass, M = ρV	kg	0.162
Q	mL/min	1.39
EBCT = V_ε_/Q	min	120
SLR = Q/A	cm/min	0.10

**Table 2:** BET Specific surface area and pore size of bone-composite.

Surface Characteristics	Values
BET surface area	251.9669 m^2^/g
External surface area	52.3858 m^2^/g
Total pore volume	0.165640 cm^3^/g
Average pore size	26.6732 A (2.66732 nm)

## Data Availability

Not applicable.

## References

[1] Adebisi G.A., Chowdhury Z.Z., Alaba P. (2017). Equilibrium, kinetic, and thermodynamic studies of lead ion and zinc ion adsorption from aqueous solution onto activated carbon prepared from palm oil mill effluent. J. Clean. Prod..

[2] Adeleke O.A., Latiff A.A.A., Saphira M.R., Daud Z., Ismail N., Ahsan A., Ab Aziz N.A., Ndah M., Kumar V., Al-Gheethi A., Ahsan A., Ismail A.F. (2019). Locally Derived Activated Carbon from Domestic, Agricultural and Industrial Wastes for the Treatment of Palm Oil Mill Effluent. Nanotechnology in Water and Wastewater Treatment.

[3] Chin M.J., Poh P.E., Tey B.T., Chan E.S., Chin K.L. (2013). Biogas from palm oil mill effluent (POME): Opportunities and challenges from Malaysia’s perspective. Renew. Sustain. Energy Rev..

[4] Mukherjee I., Sovacool B. (2014). Palm oil-based biofuels and sustainability in southeast Asia: A review of Indonesia, Malaysia, and Thailand. Renew. Sustain. Energy Rev..

[5] Adeleke A.R.O., Latiff A.A.A., Daud Z., Daud N.F.M., Aliyu M.K. (2017). Heavy Metal Removal from Wastewater of Palm Oil Mill Using Developed Activated Carbon from Coconut Shell and Cow Bones. Key Eng. Mater..

[6] Tan Y.D., Lim J.S. (2019). Feasibility of palm oil mill effluent elimination towards sustainable Malaysian palm oil industry. Renew. Sustain. Energy Rev..

[7] Som A.M., Yahya A. (2021). Kinetics and performance study of ultrasonic-assisted membrane anaerobic system using Monod Model for Palm Oil Mill Effluent (POME) treatment. Clean. Eng. Technol..

[8] Sani S., Dashti A.F., Adnan R. (2020). Applications of Fenton oxidation processes for decontamination of palm oil mill effluent: A review. Arab. J. Chem..

[9] Mohammed R.R., Chong M.F. (2014). Treatment and decolorization of biologically treated Palm Oil Mill Effluent (POME) using banana peel as novel biosorbent. J. Environ. Manag..

[10] Adeleke O.A., Latiff A.A.A., Saphira M.R., Daud Z., Ismail N., Ahsan A., Ab Aziz N.A., Al-Gheethi A., Kumar V., Fadilat A., Ahsan A., Ismail A.F. (2019). Principles and Mechanism of Adsorption for the Effective Treatment of Palm Oil Mill Effluent for Water Reuse. Nanotechnology in Water and Wastewater Treatment.

[11] Oyekanmi A.A., Latiff A.A.A., Daud Z., Mohamed R.M.S.R., Ab Aziz N.A., Ismail N., Rafatullah M., Ahmad A., Hossain K. (2019). Adsorption of pollutants from palm oil mill effluent using natural adsorbents: Optimization and isotherm studies. Desalination Water Treat..

[12] Thangalazhy-Gopakumar S., Al-Nadheri W.M.A., Jegarajan D., Sahu J.N., Mubarak N.M., Nizamuddin S. (2015). Utilization of palm oil sludge through pyrolysis for bio-oil and bio-char production. Bioresour. Technol..

[13] Ahmad A., Chong M., Bhatia S. (2007). Mathematical modeling of multiple solutes system for reverse osmosis process in palm oil mill effluent (POME) treatment. Chem. Eng. J..

[14] Bhatia S., Othman Z., Ahmad A.L. (2007). Coagulation–flocculation process for POME treatment using Moringa oleifera seeds extract: Optimization studies. Chem. Eng. J..

[15] Saeed M.O., Azizli K.A.M., Isa M.H., Ezechi E.H. (2016). Treatment of POME using Fenton oxidation process: Removal efficiency, optimization, and acidity condition. Desalination Water Treat..

[16] Charles A., Cheng C.K. (2019). Photocatalytic treatment of palm oil mill effluent by visible light-active calcium ferrite: Effects of catalyst preparation technique. J. Environ. Manag..

[17] Amat N.A., Tan Y.H., Lau W., Lai G., Ong C.S., Mokhtar N., Sani N., Ismail A.F., Goh P., Chong K. (2015). Tackling colour issue of anaerobically-treated palm oil mill effluent using membrane technology. J. Water Process. Eng..

[18] Rosli M.A., Daud Z., Ridzuan M.B., Aziz N.A.A., Awang H.B., Adeleke A.O., Hossain K., Ismail N. (2019). Equilibrium isotherm and kinetic study of the adsorption of organic pollutants of leachate by using micro peat-activated carbon composite media. Desalination Water Treat..

[19] Bulgariu L., Bulgariu D. (2018). Functionalized soy waste biomass—A novel environmental-friendly biosorbent for the removal of heavy metals from aqueous solution. J. Clean. Prod..

[20] Malakahmad A., Lahin F.A., Yee W. (2014). Biodegradation of High-Strength Palm Oil Mill Effluent (POME) through Anaerobes Partitioning in an Integrated Baffled Reactor Inoculated with Anaerobic Pond Sludge. Water Air Soil Pollut..

[21] Adeleke A.O., Latiff A.A.A., Al-Gheethi A., Daud Z. (2017). Optimization of operating parameters of novel composite adsorbent for organic pollutants removal from POME using response surface methodology. Chemosphere.

[22] Bello M., Nourouzi M., Abdullah L.C., Choong T.S., Koay Y., Keshani S. (2013). POME is treated for removal of color from biologically treated POME in fixed bed column: Applying wavelet neural network (WNN). J. Hazard. Mater..

[23] Bello M., Nourouzi M., Abdullah L. (2014). Tertiary treatment of biologically treated POME in fixed-bedC: Color and COD removal. Adv. Environ. Biol..

[24] Sirajudheen P., Karthikeyan P., Krishnapillai R., Meenakshi S. (2020). Effective removal of organic pollutants by adsorption onto chitosan supported graphene oxide-hydroxyapatite composite: A novel reusable adsorbent. J. Mol. Liq..

[25] Li P., Gao B., Li A., Yang H. (2020). Evaluation of the selective adsorption of silica-sand/anionized-starch composite for removal of dyes and Cupper(II) from their aqueous mixtures. Int. J. Biol. Macromol..

[26] Sirajudheen P., Karthikeyan P., Ramkumar K., Nisheetha P., Meenakshi S. (2021). Magnetic carbon-biomass from the seeds of Moringa oleifera@MnFe_2_O_4_ composite as an effective and recyclable adsorbent for the removal of organic pollutants from water. J. Mol. Liq..

[27] Boucherdoud A., Kherroub D.E., Bestani B., Benderdouche N., Douinat O. (2021). Fixed-bed adsorption dynamics of methylene blue from aqueous solution using alginate-activated carbon composites adsorbents. Alger. J. Environ. Sci. Technol..

[28] Edathil A.A., Pal P., Kannan P., Banat F. (2019). Total organic acid adsorption using alginate/clay hybrid composite for industrial lean amine reclamation using fixed-bed: Parametric study coupled with foaming. Int. J. Greenh. Gas Control..

[29] Wang R., Shin C.-H., Park S., Park J.-S., Kim D., Cui L., Ryu M. (2014). Removal of lead (II) from aqueous stream by chemically enhanced kapok fiber adsorption. Environ. Earth Sci..

[30] Nguyen P., Nguyen T.A., Bhandari B., Prakash S. (2016). Comparison of solid substrates to differentiate the lubrication property of dairy fluids by tribological measurement. J. Food Eng..

[31] Halim A.A., Aziz H.A., Johari M.A.M., Ariffin K.S. (2010). Comparison study of ammonia and COD adsorption on zeolite, activated carbon and composite materials in landfill leachate treatment. Desalination.

[32] Oyekanmi A.A., Latiff A.A.A., Daud Z., Daud N.M., Gani P. (2017). Adsorption of Heavy Metal from Palm Oil Mill Effluent on the Mixed Media Used for the Preparation of Composite Adsorbent. MATEC Web Conf..

[33] Gui Q., Zhang J., Hu K., Ouyang Q., Shi S., Chen X. (2020). Hydrogen bonding-induced hydrophobic assembly yields strong affinity of an adsorptive membrane for ultrafast removal of trace organic micropollutants from water. J. Mater. Chem. A.

[34] APHA (2005). Standard Methods for the Examination of Water and Wastewater.

[35] Oyekanmi A.A., Latiff A.A.A., Daud Z., Mohamed R.M.S.R., Ismail N., Ab Aziz A., Rafatullah M., Hossain K., Ahmad A., Abiodun A.K. (2019). Adsorption of cadmium and lead from palm oil mill effluent using bone-composite: Optimisation and isotherm studies. Int. J. Environ. Anal. Chem..

[36] Higai D., Huang Z., Qian E.W. (2021). Preparation and surface characteristics of phosphoric acid-activated carbon from coconut shell in air. Environ. Prog. Sustain. Energy.

[37] Tan K., Hameed B. (2017). Insight into the adsorption kinetics models for the removal of contaminants from aqueous solutions. J. Taiwan Inst. Chem. Eng..

[38] Pal S., Mukherjee S., Ghosh S. (2014). Nonlinear kinetic analysis of phenol adsorption onto peat soil. Environ. Earth Sci..

[39] Ujang Z., Buckley C. (2002). Water and wastewater in developing countries: Present reality and strategy for the future. Water Sci. Technol..

[40] Pradhan S., Boernick H., Kumar P., Mehrotra I. (2016). Removal of dissolved organic carbon by aquifer material: Correlations between column parameters, sorption isotherms and octanol-water partition coefficient. J. Environ. Manag..

[41] Gupta S.S., Bhattacharyya K.G. (2008). Immobilization of Pb(II), Cd(II) and Ni(II) ions on kaolinite and montmorillonite surfaces from aqueous medium. J. Environ. Manag..

[42] Cechinel M.A.P., de Souza S.M.A.G.U., de Souza A.A.U. (2014). Study of lead (II) adsorption onto activated carbon originating from cow bone. J. Clean. Prod..

[43] Duman O., Tunç S., Bozoğlan B.K., Polat T.G. (2016). Removal of triphenylmethane and reactive azo dyes from aqueous solution by magnetic carbon nanotube-κ-carrageenan-Fe_3_O_4_ nanocomposite. J. Alloys Compd..

[44] Nair V., Panigrahy A., Vinu R. (2014). Development of novel chitosan–lignin composites for adsorption of dyes and metal ions from wastewater. Chem. Eng. J..

[45] Leyva-Ramos R., Ovalle-Turrubiartes J., Sanchez-Castillo M. (1999). Adsorption of fluoride from aqueous solution on aluminum-impregnated carbon. Carbon.

[46] Zhang W., Honaker R., Groppo J. (2017). Flotation of monazite in the presence of calcite part I: Calcium ion effects on the adsorption of hydroxamic acid. Miner. Eng..

[47] Gutiérrez-Segura E., Colin-Cruz A., Solache-Ríos M., Fall C. (2012). Removal of Denim Blue from Aqueous Solutions by Inorganic Adsorbents in a Fixed-Bed Column. Water Air Soil Pollut..

[48] Zeng H., Arashiro M., Giammar D.E. (2008). Effects of water chemistry and flow rate on arsenate removal by adsorption to an iron oxide-based sorbent. Water Res..

[49] Yan Y., An Q., Xiao Z., Zheng W., Zhai S. (2017). Flexible core-shell/bead-like alginate@PEI with exceptional adsorption capacity, recycling performance toward batch and column sorption of Cr(VI). Chem. Eng. J..

[50] Kalavathy H., Karthik B., Miranda L.R. (2010). Removal and recovery of Ni and Zn from aqueous solution using activated carbon from Hevea brasiliensis: Batch and column studies. Colloids Surf. B Biointerfaces.

[51] Ghasemi M., Keshtkar A.R., Dabbagh R., Safdari S.J. (2011). Biosorption of uranium(VI) from aqueous solutions by Ca-pretreated *Cystoseira indica alga*: Breakthrough curves studies and modeling. J. Hazard. Mater..

[52] Alardhi S.M., Albayati T.M., Alrubaye J.M. (2020). Adsorption of the methyl green dye pollutant from aqueous solution using mesoporous materials MCM-41 in a fixed-bed column. Heliyon.

[53] Chu K.H. (2020). Breakthrough curve analysis by simplistic models of fixed bed adsorption: In defense of the century-old Bohart-Adams model. Chem. Eng. J..

[54] Ahmad A.A., Hameed B.H. (2010). Fixed-bed adsorption of reactive azo dye onto granular activated carbon prepared from waste. J. Hazard. Mater..

[55] Preetha B., Viruthagiri T. (2007). Batch and continuous biosorption of chromium(VI) by Rhizopus arrhizus. Sep. Purif. Technol..

[56] Aksu Z., Gönen F. (2004). Biosorption of phenol by immobilized activated sludge in a continuous packed bed: Prediction of breakthrough curves. Process. Biochem..

[57] Golie W.M., Upadhyayula S. (2016). Continuous fixed-bed column study for the removal of nitrate from water using chitosan/alumina composite. J. Water Process. Eng..

[58] El-Naas M.H., Abu-Alhaija M., Al-Zuhair S. (2017). Evaluation of an activated carbon packed bed for the adsorption of phenols from petroleum refinery wastewater. Environ. Sci. Pollut. Res..

